# Mining buried plant trait data from the Global Biodiversity Information Facility (GBIF): Comparison and integration with multiple plant trait databases

**DOI:** 10.1002/aps3.70083

**Published:** 2026-10-14

**Authors:** Takuro Katori, Michio Oguro

**Affiliations:** ^1^ Forestry and Forest Products Research Institute, Forest Research and Management Organization 1 Matsunosato Tsukuba 305‐8687 Ibaraki Japan

**Keywords:** flower color, GBIF, growth form, lifespan, plant height, Raunkiæran shortfall, text mining, trait

## Abstract

**Premise:**

Despite the availability of several plant trait databases, significant traits remain undocumented for a substantial number of species. To address this issue, the current study explored digital text from the Global Biodiversity Information Facility (GBIF) as an innovative source of plant trait information and evaluated its capacity to reduce existing deficiencies.

**Methods:**

Data were gathered on lifespan (*LS*), growth form (*GF*), flower color (*FC*), and maximum plant height (*PH*_*max*_) for vascular plants from GBIF and four common plant trait databases. The resulting dataset was used to evaluate the novelty and consistency of trait values derived from GBIF's digital text relative to those from other databases.

**Results:**

Incorporation of GBIF‐extracted trait data led to increases of 1.2‐, 1.2‐, 5.5‐, and 2.7‐fold in the number of species with documented *LS*, *GF*, *FC*, and *PH*_*max*_ traits, respectively. GBIF's trait data demonstrated high congruence with other databases, except in the case of *FC*. Additionally, a comprehensive dataset compiling these trait values from GBIF and the four databases was created, offering a valuable resource for future plant trait research.

**Discussion:**

Our findings suggest that digital text in GBIF represents a significant source of plant trait information and can effectively help bridge information gaps.

Traits are morphological, physiological, or phenological characteristics that can be measured at the individual level (Violle et al., 2007). The diversity and distribution patterns of traits among individuals and species are driven by abiotic and biotic environmental factors. Thus, trait data provide valuable insights into ecological functions and species' responses to environmental changes (Valladares et al., 2007; Ordoñez et al., 2009; Hoffmann and Sgrò, 2011; Foden et al., 2013), underscoring the importance of systematically compiling and publishing trait information.

A substantial body of trait data for vascular plants has been collected and published (Wright et al., 2004; eFloras, 2008; Maitner et al., 2018; Kattge et al., 2020). Recent research in plant functional ecology utilizes resources such as TRY (Kattge et al., 2011, 2020), the Botanical Information and Ecology Network (BIEN) (Maitner et al., 2018), the Rice database (Rice et al., 2019), eFloras (2008), and various scientific publications. Global‐scale analyses employing these databases have elucidated relationships between plant traits, environmental variables (Morin and Chuine, 2006; Lamanna et al., 2014; Šímová et al., 2018; Rice et al., 2019; Vallicrosa et al., 2022), and inter‐trait correlations or independence (Wright et al., 2004; Morin and Chuine, 2006; Díaz et al., 2016; Pierce et al., 2017; Shiklomanov et al., 2020).

Nevertheless, despite the existence of these plant trait databases, plant trait information remains incomplete (Maitner et al., 2022). This deficiency, referred to as the Raunkiæran shortfall (Hortal et al., 2015), limits the accuracy and scope of studies that rely on comprehensive trait coverage (Cornwell et al., 2019; Perez et al., 2019; Maitner et al., 2022). Addressing this knowledge gap is a significant challenge in the field. While trait data can be obtained from literature sources or through direct measurements of preserved or live specimens, acquiring large‐scale coverage across species is constrained by practical limitations such as time, funding, and available resources. Therefore, leveraging digitized and publicly accessible datasets that remain underutilized presents a promising approach to mitigating these limitations.

In this context, the Global Biodiversity Information Facility (GBIF) (GBIF, 2025) database represents a valuable resource for obtaining plant trait data. Historically, GBIF has primarily served as an important repository for plant distribution records (coordinates or locations) (GBIF Secretariat, 2021) and is categorized as a “distribution” database by König et al. (2019). However, GBIF also provides specimen images and associated textual metadata, which can contain extractable trait information. Although some efforts have focused on deriving plant trait data, such as leaf dimensions, from GBIF specimen images (Kommineni et al., 2021), extraction from textual metadata remains rare. Inclusion of species in the GBIF database with trait data that are not found in established trait repositories (e.g., TRY, BIEN, eFloras, and the Rice database) could help address existing gaps and support more comprehensive ecological research.

In the current study, we focused on global vascular plant species and evaluated the novelty and congruence of plant trait information extracted from GBIF's digital text against established plant trait databases. The primary aim was to determine whether GBIF text data can help address gaps in available trait information. While GBIF text contains a range of plant trait details (e.g., lifespan, growth form, flower color, plant height, leaf phenology, climbing habit, and epiphytic habit), we focused on four traits—lifespan, growth form, flower color, and plant height—due to their prevalence in GBIF text. Specifically, we investigated (1) the number of novel species for which trait data could be obtained by incorporating GBIF text alongside traditional databases (TRY, BIEN, eFloras, Rice database), and (2) the congruence of trait values extracted from GBIF in comparison with those from established sources. Congruency assessments were conducted by comparing trait values for shared species across databases for each investigated trait, using all possible database pairs. Additionally, during the analysis, a unified dataset was compiled for the four traits (lifespan, growth form, flower color, and maximum plant height) across the five databases, including GBIF. By doing so, our results will help fill gaps in trait data and support future large‐scale trait studies.

## METHODS

All data processing and analyses were performed using R software (version 4.1.1) (R Core Team, 2021), unless otherwise specified.

### Traits

Four plant traits selected for their functional and evolutionary relevance were extracted and evaluated: lifespan (*LS*), growth form (*GF*), flower color (*FC*), and maximum plant height (*PH*_*max*_). When classified by intercorrelations, plant traits can generally be categorized as size‐related or leaf economy–related (Díaz et al., 2016). Specifically, *LS*, *GF*, and *PH*_*max*_ are interrelated (Moles and Leishman, 2008; Díaz et al., 2016) and are considered size‐related traits associated with diverse functional and evolutionary processes. Such traits have been linked to mechanisms including light competition, growth cost, water transport cost (Westoby et al., 2002; Moles et al., 2009), frost tolerance (Swenson and Weiser, 2010; Boonman et al., 2020), *r‐K* selection (Díaz et al., 2016), the proportion of polyploid species (Stebbins, 1971; Rice et al., 2019), rates of molecular and ecological niche evolution (Smith and Donoghue, 2008; Smith and Beaulieu, 2009; Hawkins et al., 2011), speciation and extinction rates (Soltis et al., 2013), and range size (Morin and Chuine, 2006). In contrast, *FC* is primarily involved in pollinator attraction and is notably significant in studies on the evolution of angiosperms (Narbona et al., 2021).

Although *LS* and *GF* classifications differ across databases, we standardized trait values by reclassifying them as annual or perennial for *LS* and herbaceous or woody for *GF*. In this context, *LS* refers to the potential maximum lifespan of a species. If variable lifespans occur within a species (e.g., “annual or short‐lived perennial”), the longest possible lifespan was assigned (“perennial,” in the preceding example). Following Rice et al. (2019), we assumed that the same taxon did not exhibit both annual and woody characteristics. Perennials, however, may be either herbaceous or woody, indicating that, while related, *LS* and *GF* are not directly correlated and thus can be analyzed independently.

In this study, *FC* was designated as a trait specific to angiosperms and defined to encompass the color of the entire flower, as well as the colors of the corolla, petal, calyx, sepal, perianth, and tepal. The initial dataset preserved original flower color descriptions (e.g., pink, white‐pink, whitish pink, pale pink); these were subsequently consolidated into 11 colors (black, blue, brown, gray, green, orange, pink, purple, red, white, and yellow) for comparison across databases. Values for *PH*_*max*_ and candidate plant height (*PH*) were treated as continuous variables.

### The trait databases

The five databases used in this study exhibited varied content and structural characteristics (Tables 1 and 2). The GBIF (https://www.gbif.org/) serves as a comprehensive repository of organism occurrences worldwide. Although GBIF is primarily employed for distributional data, it also contains trait information within three specific columns (*occurrenceRemarks*, *fieldNotes*, and *eventRemarks*) of occurrence records. As these columns comprise unstructured free text, they may include non‐trait contextual details that could lead to inaccurate identification of trait values. To mitigate this issue, a filtering process was implemented, in which the associated data can be downloaded in tabular format, with each row representing an individual taxon occurrence (e.g., specimen or observation). In this study, all occurrences of vascular plants were retrieved (337,549,876 records) (GBIF.org, 2021), with each table entry regarded as a single record. Notably, GBIF includes entries in multiple languages (Table 2).

**Table 1:** Summary of the contents and coverage of the databases used in this study.

Database	Contents	Clade coverage	Geographic coverage
GBIF	Taxonomy Distribution Traits (hardly used)	All organisms	World
TRY	Traits	Plants	World
BIEN	Taxonomy Phylogeny Traits Distribution Abundance	Plants	World
Rice database	Traits (only lifespan and growth form)	Plants	World
eFloras	Taxonomy Traits Distribution	Plants	North America and China

**Table 2:** Overview of the structures of the trait databases used in this study.

Database	Format	Can there be multiple records for a taxon (species or infraspecific taxon)?	How are trait values present in each record?	Is non‐trait information mixed with trait information?	Number of traits (*LS*, *GF*, *FC*, or *PH*)^a^ in a record	Can a record contain multiple classes (for *LS*, *GF*, and *FC*)^b^ or a range (for *PH*) for a trait?	Language
GBIF	Table	Yes	In the text	Yes	0 to 4	Yes	Multiple
TRY and BIEN	Table	Yes	In most records, only trait value(s) are stored in the data field. In some records, the values are included in the text.	No	1 (*LS*, *GF*, *FC*, or *PH*) or 2 (*LS* and *GF*)	Yes^c^	English
Rice database	Table	No	Only a trait value word is stored in each data field.	No	1 (*LS* or *GF*) or 2 (*LS* and *GF*)	No	English
eFloras	Collection of description text	Yes (up to two records)^d^	In the text	Yes	0 to 4	Yes	English

Abbreviations: *FC*, flower color; *GF*, growth form; *LS*, lifespan; *PH*, plant height.

^a^ Traits refer to the traits used in this study (i.e., *LS*, *GF*, *FC*, and *PH*) rather than those defined in the original databases.

^b^ Examples include “*annual or perennial*” and “*herb or subshrub*.”

^c^ In the *PH* data for BIEN, no range values are provided.

^d^ For taxa listed in both *Flora of China* and *Flora of North America*, the number of records was counted as two.

TRY (Kattge et al., 2011) is the most widely referenced global database for plant traits. From 2007 to 2019, it was cited in over 250 publications (Kattge et al., 2020) and featured prominently in large‐scale studies on plant traits (Sandel et al., 2015; Díaz et al., 2016; Butler et al., 2017; Pierce et al., 2017; Bruelheide et al., 2018; Cornwell et al., 2019; Perez et al., 2019; Boonman et al., 2020; Shiklomanov et al., 2020; König et al., 2021). TRY data can be downloaded in tabular format, with rows representing the value(s) of a trait for a given taxon. For this analysis, a dataset containing 28,659,648 rows was compiled by selecting relevant trait names from the website (Kattge et al., 2020) (Database version 6; downloaded on 27 November 2022). Selected traits included *plant lifespan (longevity)*, *plant growth form*, *Plant life form (Raunkiaer life form)*, *plant woodiness*, *plant vegetative regeneration capacity*, and *plant functional type (PFT)* for *LS* and *GF*; *flower color*, *flower calyx color*, and *flower corolla color* for *FC*; and *plant height vegetative* and *plant height generative* for *PH*.

BIEN is a recently developed database encompassing taxonomic, phylogenetic, trait, distribution, and abundance information for plants worldwide (Maitner et al., 2018). It has contributed to large‐scale plant trait studies (Kelly et al., 2021; Kling and Ackerly, 2021) and shares a similar structure with TRY. By specifying trait names using the *BIEN_trait_trait* function in the R package BIEN version 1.2.4 (accessed 16 November 2022), we downloaded a table with 10,619,951 rows. Traits specified included *longest whole plant longevity*, *maximum whole plant longevity*, *whole plant growth form*, *whole plant growth form diversity*, and *whole plant woodiness* for *LS* and *GF*; *flower color* for *FC*; and *maximum whole plant height* and *whole plant height* for *PH*.

The Rice database (“Lifeform database”; Rice et al., 2019) provides a comprehensive list of *LS* and *GF* for plants worldwide. It aggregates information from several sources (i.e., eFloras [http://www.efloras.org/], Encyclopedia of Life [Parr et al., 2014], Index to Chromosome Numbers in Asteraceae [Semple and Watanabe, 2023], International Legume Database & Information Service [https://www.ildis.org/], the Missouri Botanical Garden's Tropicos database [https://www.tropicos.org/], World Checklist of Selected Plant Families [website merged with Plants of the World Online in 2022 (POWO, 2026)], and the database compiled by Zanne et al. [2014]) and has been used in large‐scale plant trait studies (Poppenwimer et al., 2023; Wang et al., 2023). This database was accessed via the supplementary materials published by Rice et al. (2019).

eFloras (http://www.efloras.org/) is an online resource providing flora descriptions—including taxonomy, traits, and distributions—from various regions. It has been widely used in large‐scale trait studies (Thomson et al., 2011; Chen et al., 2019). For the current study, only the *Flora of North America* and *Flora of China* were used, as these sections are available in machine‐readable HTML format, whereas other sections are presented as PDFs, which are less suitable for automated data extraction. Taxon descriptions were downloaded through web scraping using the R package rvest version 1.0.2 (26 August 2023). Despite the inclusion of eFloras‐derived trait data in the Rice database, eFloras was also directly accessed to augment trait value coverage. The rationale for this approach is that the Rice database extracts only trait values explicitly described at the species or infraspecific levels, whereas eFloras often presents shared traits at higher taxonomic levels. Supplementing the dataset with trait information from higher taxonomic categories enhances completeness (Coleman et al., 2023). Accordingly, data were collected not only for species and infraspecific taxa but also for broader groups such as families, genera, and sections.

### Standardization of taxon names

Taxon names (species or infraspecific taxa) were extracted from each record within their respective databases. The sources for extraction included the *acceptedScientificName* column in GBIF, the *AccSpeciesName* column in TRY, the *scrubbed_species_binomial* column in BIEN, the *Species* column in the Rice database, and the headings of each description in eFloras. To standardize synonyms across taxon names, the Leipzig Catalogue of Vascular Plants (LCVP) was used (Freiberg et al., 2020). Standardization was conducted using the *lcvp_search* function from the R package lcvplants (version 2.0.0; Freiberg et al., 2020), which generated the “Output.Taxon” field; author names were subsequently removed. If “Output.Taxon” referred to an infraspecific name, it was modified to its corresponding binomial (species) name by removing the infraspecific component. Records where “Output.Taxon” referred to a taxonomic rank higher than species (e.g., genus) or NA values were excluded from the dataset; however, for eFloras, records referring to a taxonomic rank higher than species were retained because they could be used to assign trait values to taxa at lower taxonomic ranks (as described in detail below).

### Extracting trait values from each record

For databases other than the Rice database, records can contain multiple candidate values for *LS*, *GF*, and *FC* (Table 2). Therefore, we extracted all candidate values for these traits from each vascular plant record across all databases. In contrast, each record in all databases contains only one entry for *PH*, which appears as either a single value or a range. Sources for these trait values in each database are detailed in Appendix S1 (see Supporting Information). If records in GBIF, TRY, or BIEN lacked source information, they were excluded. Before extracting trait values, we converted all letters and numbers to half‐width, lowercase letters without accent marks.

#### GBIF and eFloras

In GBIF, multiple records may refer to the same specimen or observation; thus, duplicates were removed by retaining only one record per species when identical *catalogNumber* values appeared. Records with blank *catalogNumber* fields were retained.

Trait information in GBIF and eFloras is embedded within descriptive text associated with occurrences or taxonomic groups, requiring us to process the text to identify and assign candidate trait values. These texts often contain expressions that can lead to false trait detections (e.g., “annual rainfall” should not be interpreted as “annual” for *LS*). To address this, such misleading expressions were removed from all GBIF and eFloras records before trait values were extracted. First, a convenience sample of record texts was manually reviewed to identify (1) expressions to exclude and (2) expressions indicating trait values, generating an initial removal list and trait‐expression list. There was no fixed number of records for this sampling. For unfamiliar languages, Google Translate (https://translate.google.com/) was used to interpret the text. Online resources were also consulted to determine common trait‐related expressions in specific languages, and plausible alternative phrasings present in the dataset were added via corpus‐wide search. Second, all expressions on the removal list were deleted from each record's text, after which trait values were extracted using the trait‐expression list. Third, a random sample of 500 records, each with and without extracted trait information (totaling 1000), was manually reviewed to assess extraction accuracy. If less than 1% of the records contained extraction errors (e.g., misidentifying non‐trait expressions or missing valid trait expressions), both lists were accepted. If not, problematic expressions were added to the respective lists, and steps 2–3 were repeated until error rates fell below 1%. This process was applied to each trait in GBIF and eFloras. For *PH*, to simplify patterns, any mathematical operators (>, <, and ±) and approximate qualifiers immediately preceding numbers (e.g., “ca.”; all expressions are listed in Appendix S2) were removed before starting this extraction process.

After finalizing the lists, English descriptors for flower part colors (corolla, petal, calyx, sepal, perianth, and tepal) were incorporated into the trait‐expression list for *FC* to align with its definition. The comprehensive lists detailing the removal and trait‐expression entries for each trait across each database are provided as Appendices S3–S8 (for *LS*, *GF*, and *PH*) and on Zenodo (for trait‐expression lists of *FC*; see Data Availability Statement). Note that no expressions for removal were identified during list construction for *FC*.

Although GBIF text data span multiple languages, automated translation was not integrated into processing to avoid introducing errors from machine translation. Instead, the final GBIF lists encompass expressions in all encountered languages during list construction—including English, Spanish, Portuguese, French, German, Chinese, and Japanese. The coverage of expressions varies across languages; those less commonly represented in the records may have limited expression coverage.

Descriptions of shared traits for a taxonomic group generally appear under the group's entry rather than in the description(s) of each subtaxon in the flora (Coleman et al., 2023), such as eFloras. Therefore, for eFloras, if *LS* or *GF* values were missing for a species, we extracted candidate values from higher taxonomic records (family, genus, section) in the same flora. However, this approach was not applied to *FC* or *PH* because their variation within taxa (polymorphism for *FC* and continuous variation for *PH*) violates the trait monomorphism assumption required for reliable value propagation from higher taxa (Coleman et al., 2023).

#### TRY, BIEN, and Rice databases

The extraction of *LS*, *GF*, and *FC* values from the TRY database was accomplished using specific combinations of entries within the three columns (*TraitName*, *DataName*, and *OrigValueStr*). By referencing these columns, trait values were systematically assigned to each record; details regarding these combinations and corresponding trait values are listed in Appendix S9. For *PH* values within TRY, data and units were sourced from the *StdValue*, *OrigValueStr*, and *UnitName* columns. As *OrigValueStr* merges value and unit (m or cm), whereas *StdValue* and *UnitName* delineate these elements, compilation was facilitated by prioritizing *StdValue* and *UnitName*. Notably, *PH* values in TRY may occasionally appear as ranges.

Similarly, *LS*, *GF*, and *FC* values from BIEN were extracted and assigned based on combinations from the *trait_name* and *trait_value* columns (Appendix S10). *PH* values in BIEN were obtained directly from the *trait_value* column. As *LS* and *GF* values in the Rice database align closely with the classification methods utilized in this study, no modifications were made. Unresolved values (*mixed*, *conflict*, and *NA*) were regarded as missing data.

### Construction of record‐level datasets for each trait for each database

Excluding the Rice database, individual records may contain multiple values (or value ranges) for a given trait; therefore, we implemented the following procedure to determine a representative value for each trait within each record. After identifying the candidate values for *LS* and *GF* in every record across all databases, we selected the final values for these traits. Adopting the approach of Rice et al. (2019), we considered only the following *LS* and *GF* combinations for each species: annual‐herb, perennial‐herb, and perennial‐wood. Conflicting entries and missing data for *LS* and *GF* were resolved through the following steps: (1) If a record included both annual and wood values, *LS* and *GF* were set as missing, and no further steps were applied. (2) If herb and wood values were both present, *GF* was regarded as missing, and step 3 was omitted. (3) If wood was among the values, perennial was assigned as the final *LS*. (4) Where annual and perennial values co‐occurred, perennial was prioritized for *LS*, representing the species' maximum potential *LS* in this study, and step 5 was omitted. (5) For records showing only an annual value without a corresponding perennial, *GF* was classified as an herb. For any remaining records not addressed by these criteria, the existing trait values were directly used as representative values.

For *FC*, due to variation in color depending on the flower part, if multiple‐word *FC*s (e.g., “pale pink”, “white‐pink”, “pinkish white”, “pink and white”, and “white and pink”) appeared within one record, we did not select a single color but retained the raw *FC* data as representative. Records containing distinct color descriptions for separate parts of the flower were regarded as missing values.

For *PH*, if values were reported as ranges in GBIF, eFloras, or TRY (using symbols such as “*−*”, “*~*”, “a”, or “to”; e.g., “1–2 m”, and “1m–2 m”), the upper bound was recorded as the *PH* value. All measurements were standardized to meters. The selected *PH* values underwent further cleaning to reduce the influence of outliers, excluding those exceeding known maximums for major clades based on the literature: 20 m for ferns (Wee et al., 2014), 61 m for monocots (Bernal et al., 2018), 116.07 m for *Sequoia sempervirens* (Sillett et al., 2021), and 100.8 m for other plants (Shenkin et al., 2019; Sillett et al., 2021). Clade assignment for each species (other than *Sequoia sempervirens*) was determined by appending order names during taxonomic standardization (following LCVP) (Freiberg et al., 2020), followed by class names using the GBIF species‐matching tool (https://www.gbif.org/tools/species-lookup). We classified Liliopsida as monocots, Polypodiopsida and Lycopodiopsida as ferns, and other classes as other plants. The occurrence‐level trait datasets derived from GBIF following the above procedures have been deposited on Zenodo (see Data Availability Statement).

### Construction of species‐level datasets for each trait across databases

Databases often contain multiple records for a single species (Table 2), and taxonomic name standardization may result in different species being merged. Thus, a given trait for a species within a database can have multiple records with varying values. Using the previously generated record‐level datasets, we determined a representative value for each species–trait database combination.

#### GBIF, TRY, and BIEN

For *LS*, *GF*, and *FC*, we selected the class most frequently recorded for each trait (annual or perennial for *LS*, herb or wood for *GF*, various colors for *FC*) within each database. If a tie occurred between classes with the highest frequency, the representative value was marked as missing for that species in the respective database. For *FC*, due to the presence of multilingual and multi‐color entries at the record level, generating a species‐level dataset directly from the original values was not feasible. Therefore, we focused exclusively on records denoting a single color, reclassifying them into one of 11 standard categories: black, blue, brown, gray, green, orange, pink, purple, red, white, and yellow. We then calculated the representative *FC* for each species using only standardized single‐color records. The correspondence table for color standardization has been uploaded to Zenodo (see Data Availability Statement).

For *PH*, outlier removal was initially performed at the species level within each database by iteratively applying the Smirnov–Grubbs test (Grubbs, 1969) until no outliers remained. The maximum remaining *PH* value (*PH*_*max*_) for each species served as its representative trait value. To further ensure data integrity, an additional round of outlier removal was conducted at the genus level for *PH*_*max*_ within each database, again using the Smirnov–Grubbs test (Grubbs, 1969) until no outliers remained.

#### Rice database and eFloras

The Rice database and eFloras provide a limited number of records for each species; specifically, only one record was available per taxon in the Rice database, and one or two records were available in eFloras (Table 2). Hence, species with multiple classes of candidate values for *LS*, *GF*, or *FC* were excluded from the dataset for those respective traits. For *PH*_*max*_, a representative value was assigned by calculating the maximum *PH* observed within the records for each species in each database, including all data points without excluding outliers.

### Novelty of plant traits in GBIF

The novelty of trait values sourced from GBIF was assessed by enumerating species for which trait data could be obtained exclusively from GBIF but were missing from the other four databases: TRY, BIEN, Rice database, and eFloras. To facilitate this analysis, all accepted vascular plant species—375,005 in total (as per *tab_lcvp* from the R package LCVP, version 3.0.1, dated 8 November 2022)—were categorized into four groups using the species‐level datasets constructed previously. The groups included: (a) species with focal trait values available only in databases other than GBIF, (b) species with focal trait values in GBIF and other databases, (c) species with focal trait values exclusive to GBIF, and (d) species lacking focal trait values across all databases (a + b + c + d = 375,005). Analysis was performed separately for each trait.

For comparative purposes, cases considering all four databases, collectively and individually, were prepared. The novelty of GBIF, defined as its potential to address gaps in trait information, was evaluated by comparing the proportion of species with trait values in databases other than GBIF to the total number of species ((a + b)/375,005), and the proportion of species with trait values accessible in all databases, including GBIF ((a + b + c)/375,005), calculated for each trait. Notably, because *FC* encompasses only angiosperms, the denominator for *FC* analyses was set to 359,418 species rather than 375,005.

### Congruency of plant traits in GBIF with other databases

To assess the quality of trait values extracted from GBIF relative to other databases, we investigated the degree of congruence between databases for traits assigned to the same species. Congruency indices were defined and calculated for each pairwise combination among the five databases and for each trait, using the species‐level datasets outlined previously. For *LS*, *GF*, and *FC*, the congruency index was measured as the proportion of species exhibiting identical trait values across the two compared databases (e.g., among 15,856 species with *LS* data in both GBIF and eFloras, 15,856 showed matching *LS* classes, resulting in a congruency ratio of 0.949). For *PH*_*max*_, the congruency index was defined as the mean absolute value of the log_10_ *height ratio*, with the *height ratio* calculated as the ratio of *PH*_*max*_ for the same species between two databases (e.g., for *Thalictrum javanicum*, with a *PH*_*max*_ of 1.2 m in GBIF and 1.0 m in eFloras, the absolute log_10_ *height ratio* is ~0.079). Notably, a lower congruency index for *PH*_*max*_ indicates greater similarity between the databases for this trait.

### Removal of species with suspicious representative values

To assess whether excluding species with potentially inaccurate trait values could improve the congruency of trait data across databases, we established specific criteria for species removal and conducted corresponding sensitivity analyses. Species were excluded based on the proportion of records supporting a representative value for each trait; those with proportions below set thresholds were removed. For *LS* and *GF*, threshold values ranged from 0.5 to 1 (specifically, 0.5, 0.6, 0.7, 0.8, 0.9, and 1.0). For *FC*, the threshold included 0.0, 0.2, 0.4, 0.6, 0.8, and 1.0, reflecting its criterion range (>0 and ≤1). Note that the total number of records with *FC* data, including multi‐color records, was used as the denominator for the *FC* criterion. For *PH*_*max*_, the criterion was the number of records containing *PH* values for a given species, with thresholds set at 1, 2, 3, and 4. The minimum threshold values for each criterion (0.5 for *LS* and *GF*, 0.0 for *FC*, and 1 for *PH*_*max*_) indicated that no species were removed under these conditions.

## RESULTS

### Number of species in each database and the novelty of GBIF

The databases with the largest number of species were the Rice database for *LS*, the TRY database for *GF*, and the GBIF database for *FC* and *PH*_*max*_ (Table 3, Figure 1). Species with trait values exclusively sourced from GBIF and not available in other databases were identified (Figure 2). Without removing species with suspicious representative values, the number of species solely obtained from GBIF was 38,029 for *LS*, 41,166 for *GF*, 99,101 for *FC*, and 93,709 for *PH*_*max*_ (“All other databases vs. GBIF” in Figure 2). By incorporating these GBIF‐exclusive species with those from the other four databases, the proportion of accepted vascular plants (for *FC*, angiosperms) for which trait values could be obtained increased significantly from 54% to 64% (1.2‐fold) for *LS*, 57% to 68% (1.2‐fold) for *GF*, 6% to 33% (5.5‐fold) for *FC*, and 15% to 40% (2.7‐fold) for *PH*_*max*_. Moreover, applying the maximum threshold values for excluding species did not substantially reduce GBIF‐only species counts for *LS* or *GF*; however, significant decreases were observed for *FC* and *PH*_*max*_ (Figure 2, Appendix S11).

**Table 3:** The number of species with trait values for each database and each trait.^a^

Database	Lifespan	Growth form	Flower color	Maximum plant height
GBIF	131,895 (0.35)	149,278 (0.4)	111,107 (0.31)	125,673 (0.34)
TRY	85,042 (0.23)	158,384 (0.42)	10,127 (0.03)	31,395 (0.08)
BIEN	47,137 (0.13)	93,329 (0.25)	2507 (0.01)	7896 (0.02)
Rice database	166,855 (0.44)	138,580 (0.37)	0 (0)	0 (0)
eFloras	30,269 (0.08)	30,862 (0.08)	10,364 (0.03)	30,223 (0.08)
All databases	241,056 (0.64)	253,472 (0.68)	119,522 (0.33)	148,862 (0.4)

^a^ Values in parentheses are the proportion to the accepted species number of vascular plants (375,005, based on *tab_lcvp* data in the R package LCVP version 3.0.1). However, for flower color, the calculations were based on angiosperms only (359,418).

**Figure 1:**
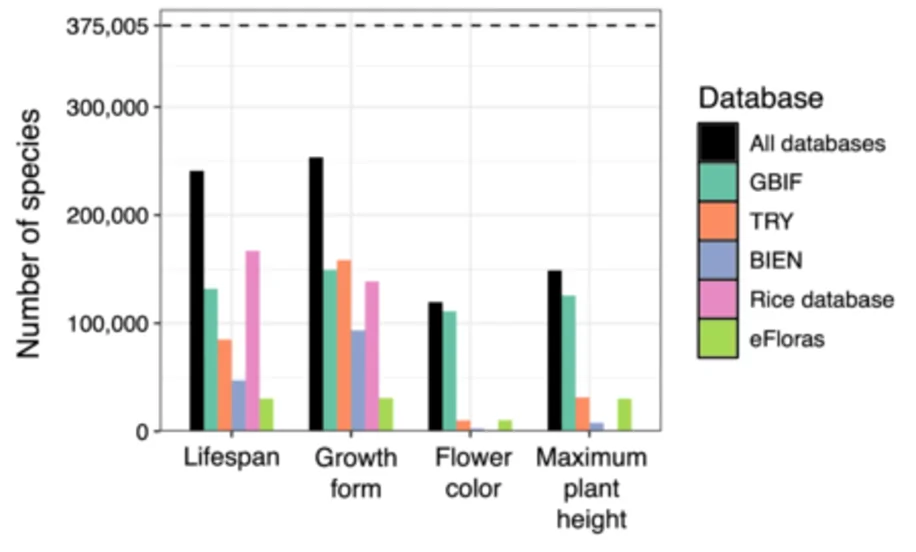
The number of species with trait values for each database and each trait (see Table 3). The dotted line represents the number of all described vascular plant species (all accepted species in *tab_lcvp* data in the R package LCVP version 3.0.1).

**Figure 2:**
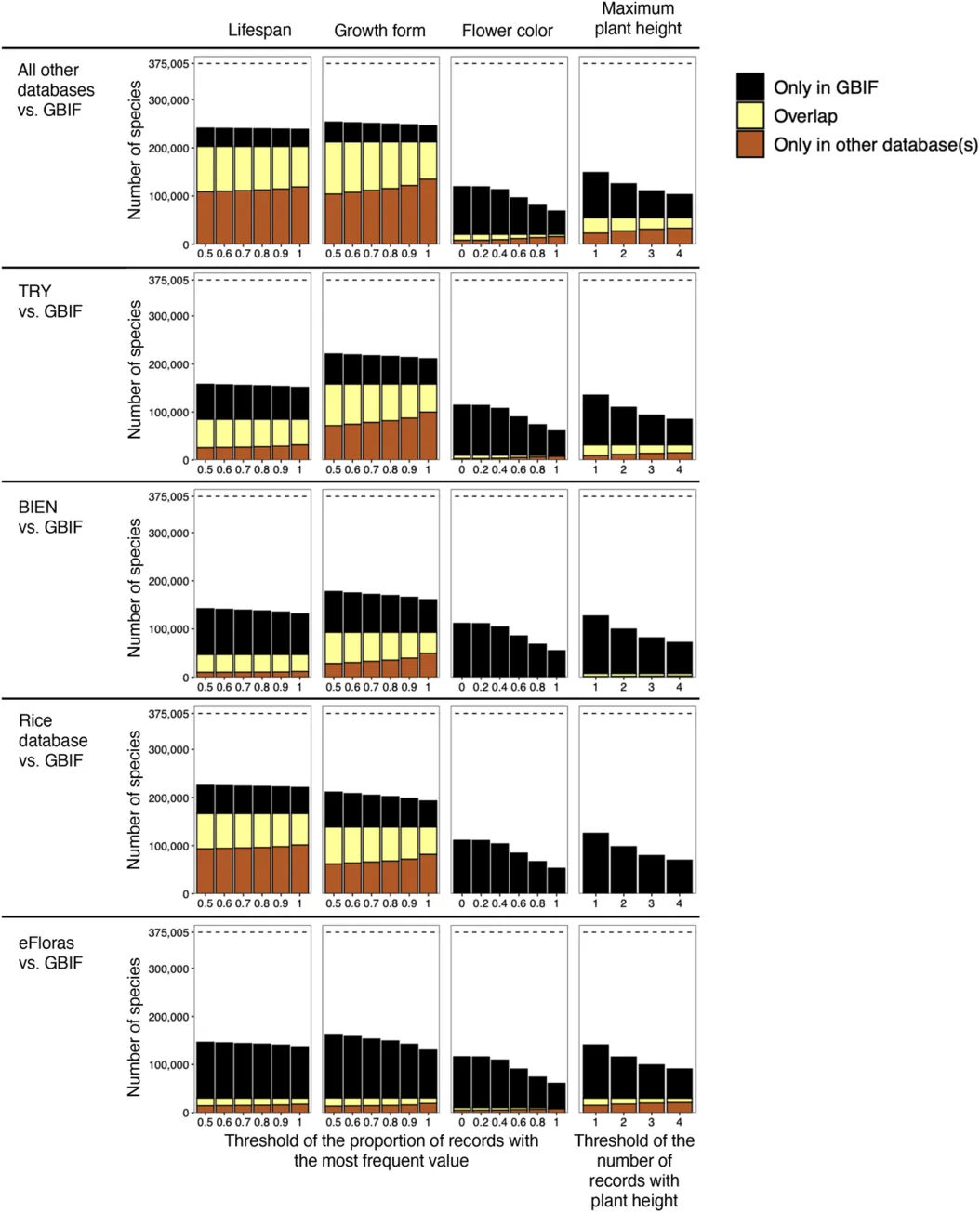
Changes in the number of species with lifespan, growth form, flower color, and maximum plant height data obtained exclusively from GBIF, other database(s), or both, following the removal of species with potentially incorrect values. The *x*‐axes represent the threshold criteria used to remove species in GBIF. Species with criterion values below the threshold were removed (i.e., higher *x*‐axis values indicate the removal of more species from GBIF). The minimum threshold values (0.5 for lifespan and growth form, 0 for flower color, and 1 for maximum plant height) indicate cases in which no species were removed. Labels: *Only in GBIF*: species unique to GBIF; *Overlap*: species found in GBIF and other databases; *Only in other database(s)*: species unique to other database(s). The total number of species in these three parts (= Only in GBIF + Overlap + Only in other databases) at the minimum threshold values are the same as the number of species of *All databases* in Figure 1. The dotted line represents the number of all described vascular plant species (all accepted species in *tab_lcvp* data in the R package LCVP version 3.0.1).

### Congruency of plant traits between databases

Without removing species with potentially erroneous values as defined by specific criteria, the congruency of trait values for identical species between GBIF and other databases was evaluated. For *LS*, BIEN exhibited the highest congruence with GBIF, while eFloras showed the lowest, with proportions of congruent species ranging from 94.9% to 99.5% (Figure 3A). For *GF*, *FC*, and *PH*_*max*_, eFloras demonstrated the greatest congruency with GBIF, whereas TRY showed the least (Figure 3B–D). The congruency proportions varied from 91.4% to 94.9% for *GF* and from 55.7% to 73.0% for *FC*. The mean absolute log_10_ (height ratio) values for *PH*_*max*_ ranged from 0.286 to 0.239. As the threshold for species removal increased, congruency between GBIF and other databases generally improved across all traits but declined for *GF* and *FC* at the maximum threshold (i.e., when only species with uniform values across all records were included; Figure 3).

**Figure 3:**
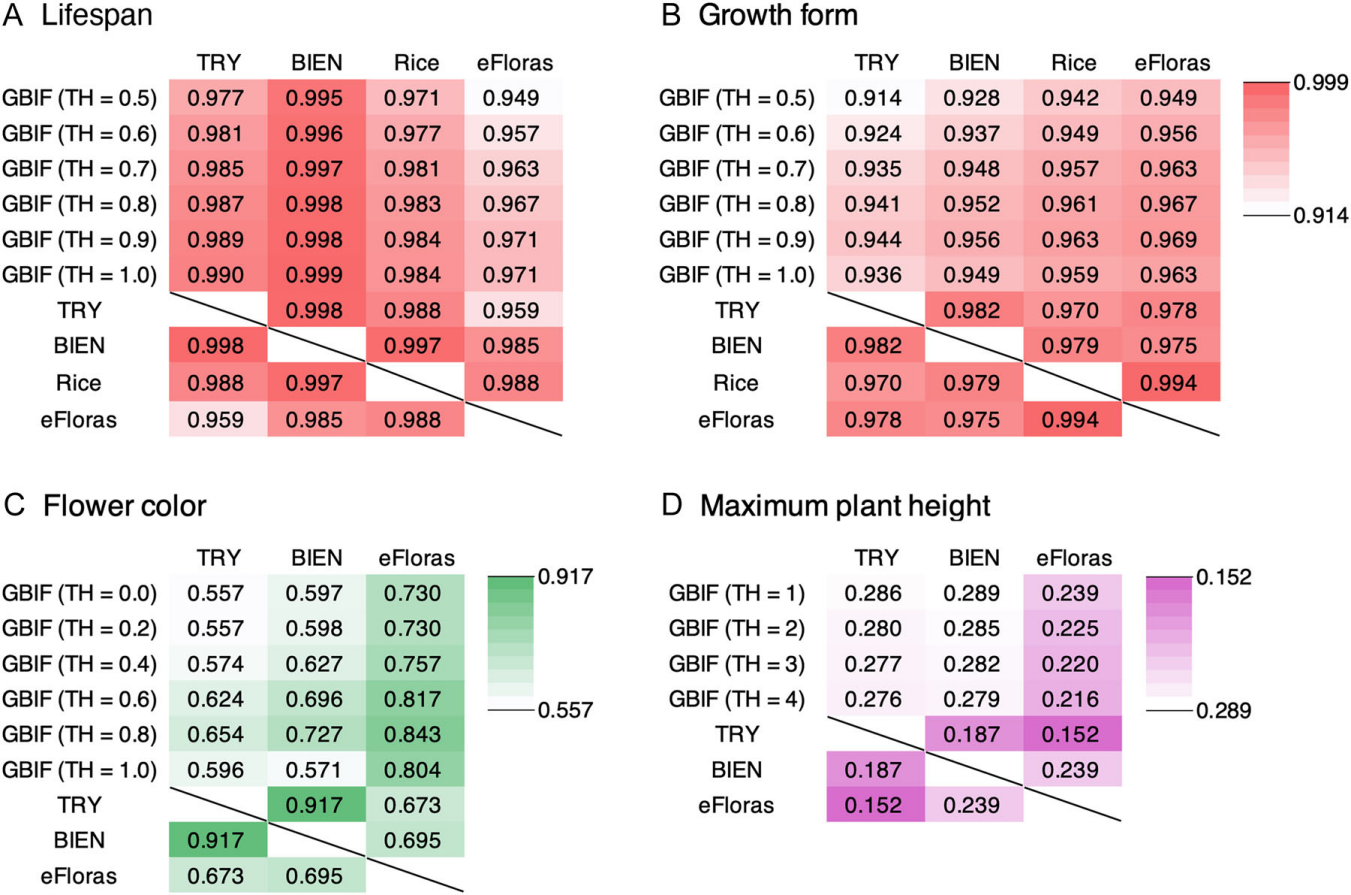
Congruency of lifespan, growth form, flower color, and maximum plant height between databases. TH, threshold values (equivalent to the threshold values in Figure 2); darker colors signify increased congruence of trait values between the same species in the two databases. Values in (A), (B), and (C) represent the proportion of species with congruent trait values between the same species in two databases. Values in (D) represent the mean absolute value of log_10_ (*height ratio*), which is the ratio of maximum plant height between the same species in two databases.

## DISCUSSION

### GBIF digital text as a new source for plant traits

Many species feature data on *LS*, *GF*, *FC*, and *PH*_*max*_ in GBIF digital text that are missing from common plant trait databases. Notably, the number of species with data for *FC* and *PH*_*max*_ significantly increased when data from the GBIF digital text were added. Generally, the traits extracted from GBIF aligned well with those from other databases, whereas congruency for *FC* was lower. To our knowledge, this is the first study to extract plant trait information directly from GBIF's digital text records. Accordingly, we propose that GBIF digital text offers a unique and valuable source of plant trait data. This new resource could help mitigate the severe Raunkiæran shortfall—the scarcity of trait information—and reduce barriers to plant trait research. In addition to database mining, researchers typically acquire missing trait details through manual literature review or direct measurement from living specimens, both of which demand considerable time and effort. Thus, acquiring substantial new trait data from GBIF's digital texts represents a significant milestone. Furthermore, each GBIF record (occurrence) may contain geographic coordinates and location data. By integrating trait and location information, geographical variations in traits can be investigated—for example, analyzing within‐species differences in *PH*.

In this study, we extracted *LS*, *GF*, *FC*, and *PH*_*max*_ data from GBIF and common plant trait databases (TRY, BIEN, Rice database, and eFloras), organizing them at the species level. This resulted in the largest comprehensive table for these traits to date (see Data Availability Statement), offering a valuable resource for future plant trait research. While previous studies have utilized data extracted from trait databases (Thomson et al., 2011; Díaz et al., 2016; Kling and Ackerly, 2021; Poppenwimer et al., 2023), consolidating information into a single table appears to streamline research processes.

### Congruency of trait values across databases

Trait data for the same species were generally consistent across GBIF and other databases, except for *FC*. The congruency observed for *FC* was marginally lower, potentially reflecting the considerable number of *FC* classes. As the criteria for species removal became more stringent, congruency between GBIF and other databases tended to increase for all traits; however, for *GF* and *FC*, it declined at the maximum threshold (i.e., when only species for which all records had the same value were included). Enhanced congruency with increasing thresholds likely resulted from the exclusion of species with erroneous records or intraspecific trait variation. Conversely, the reduced congruency for *GF* and *FC* under the strictest criteria may be due to species retained at this stage typically having fewer records; in contrast, species with many records are less likely to exhibit identical values, thereby reducing the accuracy of species‐level estimates. Thus, the interplay between minimizing erroneous records and the tendency to retain species with limited data under stricter thresholds might account for the observed trends.

### Reasons why trait information in GBIF's digital text has not been used

Despite GBIF's digital text containing plant trait information for numerous species, such data have seldom been extracted or utilized. Two factors may contribute to this underuse. First, GBIF is predominantly regarded as a resource for species distribution data (König et al., 2019), which may lead researchers to overlook the available trait information within text records. Second, trait values in the digital text are generally embedded in sentences rather than presented as isolated words or phrases (Table 2). This format renders trait information less accessible, necessitating additional steps such as removing irrelevant phrases and extracting pertinent trait information from sentences.

### Study limitations

Although quality control based on sampling indicates that our extraction method yields less than 1% false detections, some trait values may be overlooked. Consequently, while GBIF text extraction may increase the availability of data for these four traits, its novelty may be underestimated due to potential missed records. Furthermore, the trait‐extraction process depended on opportunistically developed lists for expression removal and identification, which may have resulted in variable coverage across different languages. To enhance comprehensiveness, future efforts should emphasize systematic curation of multilingual expression lists and integration of automated machine translation workflows.

### Roadmap for building a more comprehensive plant trait dataset

While this study focused on extracting only *LS*, *GF*, *FC*, and *PH*_*max*_ from GBIF digital text, our analysis revealed that these texts often include additional valuable trait information, such as leaf habit (evergreen or deciduous), climbing habit (climber or erect), and epiphytic habit (epiphyte or geophyte) (Table 4). These traits are critical for advancing the understanding of plant growth and phenology (Rowe and Speck, 2005; van Ommen Kloeke et al., 2012; Gianoli, 2015; Zotz, 2016); therefore, applying similar extraction methodologies to these data is an important direction for future research.

**Table 4:** Utilization status of plant trait information included in GBIF.

Type of data field	Plant trait	Extracted/unextracted status
Digital text (columns: *occurrenceRemarks*, *fieldNotes*, and *eventRemarks*)	*LS*, *GF*, *FC*, and *PH*	Extracted in this study
	Leaf habit (evergreen or deciduous), climbing habit (climber or erect), epiphytic habit (epiphyte or geophyte), etc.	Unextracted
Plant body in specimen images	Leaf size, leaf structure, and leaf shape	Extracted by Younis et al. (2018); Kommineni et al. (2021)
Label text in specimen images	*LS*, *GF*, and *PH*	Unextracted^a^

Abbreviations: *FC*, flower color; *GF*, growth form; *LS*, lifespan; *PH*, plant height.

^a^ Label text is also transcribed into digital text. The trait data from such textual data were extracted in this study.

Recent studies have sought to extract plant trait information from taxonomic literature (Coleman et al., 2023; Folk et al., 2024). By applying these methods, it may be possible to extract data on a broader range of species and trait types from the GBIF digital text than was achieved in the current study. Beyond digital text, GBIF also hosts plant trait data within specimen image records. Trait measurements can be derived from both the plant parts depicted and their corresponding labels. Previous studies have demonstrated the feasibility of obtaining leaf size and shape data from such images (Younis et al., 2018; Kommineni et al., 2021). While some label information has been transcribed into digital text and was included in our extracted dataset, other labels remain undigitized, resulting in incomplete data capture. Efforts to digitize label information are ongoing (Greenberg and Klas, 2008; Kirchhoff et al., 2018; Nelson and Ellis, 2019; Owen et al., 2020; Walton et al., 2020; Albani Rocchetti et al., 2021; Weaver et al., 2023; Folk et al., 2024), and future progress in this area will further augment the uniqueness and utility of GBIF's textual resources.

To establish a more comprehensive plant trait dataset—encompassing a broader range of species and traits—it may be necessary to expand beyond the five databases used in this study. Additional resources such as the Plant DNA C‐values Database (https://cvalues.science.kew.org), USDA PLANTS Database (https://plants.sc.egov.usda.gov/), Jepson eFlora (https://ucjeps.berkeley.edu/eflora/), Angiosperm Phylogeny Website (http://www.mobot.org/MOBOT/research/Apweb/), AusTraits (https://austraits.org/; Falster et al., 2021), Plant Trait Ontology (https://bioportal.bioontology.org/ontologies/PTO), BROT (https://www.uv.es/jgpausas/brot.htm; Tavsanoglu and Pausas, 2018), regional floras, and trait‐focused literature should be considered. Notably, leaf economy–related traits (e.g., leaf mass per area and leaf nitrogen content) are currently unavailable from GBIF. For species lacking such data in the other four databases, supplementary sources and methods—including direct extraction from literature and destructive sampling of specimens or living plants (Heberling, 2022)—may be required. Finally, given that GBIF includes records of organisms other than plants, it is plausible that associated digital texts, specimen images, and specimen label images hold additional useful trait data relevant to non‐plant taxa.

### Concluding remarks

The findings of this study demonstrate that GBIF text data offer a valuable new source for plant trait information, helping to fill existing gaps and support ongoing research. Integrating GBIF trait data increased the number of species with recorded trait values by factors of 1.2, 1.2, 5.5, and 2.7 for *LS*, *GF*, *FC*, and *PH*_*max*_, respectively. To achieve these results, we extracted and organized *LS*, *GF*, *FC*, and *PH*_*max*_ data from five databases, including GBIF, generating the largest dataset to date for these traits. Integrating this resource with phylogenetic, distributional, and climatic data can help clarify how geography and climate affect trait evolution and trait‐dependent speciation or extinction, which is key to biogeography and evolutionary biology. Additionally, it may facilitate the identification of species vulnerable to climate change.

## AUTHOR CONTRIBUTIONS

T.K. conceptualized the objectives and methodology of this study. M.O. refined the methodology. T.K. and M.O. conducted the analyses, wrote the manuscript, and approved the final version of the manuscript.

## Supporting information


**Appendix S1:** Sources of trait information for each trait and database.
**Appendix S2:** List of expressions representing approximations excluded prior to the extraction of plant height values.
**Appendix S3:** Expressions excluded prior to the extraction of values for lifespan and growth form.
**Appendix S4:** Expressions representing lifespan (*LS*) and growth form (*GF*) used to extract trait values from the text of GBIF and eFloras.
**Appendix S5:** Patterns of expression that could potentially cause false detection of plant height values that were removed from the records in GBIF and eFloras.
**Appendix S6:** Patterns of expression used to extract values of plant height (*PH*) from GBIF and eFloras.
**Appendix S7:** Units used for plant height extraction.
**Appendix S8:** Expressions representing the whole plant body for the extraction of *PH* from GBIF (see Appendix S6).
**Appendix S9:** Combinations of values in the *TraitNames*, *DataName*, and *OrigValueStr* columns used to assign candidate values of lifespan (*LS*) and growth form (*GF*) for each record in TRY.
**Appendix S10:** Combinations of values in the *trait_name* and *trait_value* columns used to assign candidate values of lifespan (*LS*) and growth form (*GF*) for each record in BIEN.
**Appendix S11:** Number of unique and overlapping species when comparing GBIF with four other databases (the number of species shown in Figure 2).

## Data Availability

The data associated with this study are available on Zenodo (https://doi.org/10.5281/zenodo.19130722) and include: a raw table presenting the all‐occurrence data on vascular plants downloaded from GBIF, containing the five columns used in this study as well as latitude and longitude data (File 1); text and values of *LS*, *GF*, *FC*, *PH*, latitude, and longitude extracted from each occurrence on GBIF (Files 2–4); a species‐level dataset of *LS*, *GF*, *FC*, and *PH*_*max*_ for five databases (GBIF, TRY, BIEN, Rice database, and eFloras) (File 5); and files relating to expressions used to extract *FC* from GBIF and eFloras (Files 6–8).
