# Supplementary material for: Mining buried plant trait data from the Global Biodiversity Information Facility (GBIF): Comparison and integration with multiple plant trait databases
Source: Appl Plant Sci. 2026 Oct 14:e70083. Online ahead of print. doi: 10.1002/aps3.70083 (PMC13662890; doi:10.1002/aps3.70083)
Supplement: Supplementary file 1 [file APS3-9999-e70083-s001.docx]

**Supplementary information on methods used for extracting trait values, and the number of species shown in Figure 2.**

**Appendix S1**. Sources of trait information for each trait and database.

| **Database** | **Trait** | **Source(s) of trait information** |
| --- | --- | --- |
| GBIF | *LS*, *GF*, *FC*, and *PH* | *occurrenceRemarks* column: notes on the occurrence |
|  |  | *fieldNotes* column: field note |
|  |  | *eventRemarks* column: notes on the event |
|  |  |  |
| TRY | *LS*, *GF*, and *FC* | *TraitName* column: name of trait |
|  |  | *DataName* column: name of specific trait or context information |
|  |  | *OrigValueStr* column: original value as text string |
|  |  |  |
|  | *PH* | *TraitName* column: name of trait |
|  |  | *OrigValueStr* column: original value as text string |
|  |  | *StdValue* column: standardized value |
|  |  | *UnitName* column: unit name |
|  |  |  |
| BIEN | *LS*, *GF*, *FC*, and *PH* | *trait_name* column |
|  |  | *trait_value* column |
|  |  |  |
| Rice database | *LS* | *Life_cycle* column |
|  |  |  |
|  | *GF* | *Growth_form* column |
|  |  |  |
| eFloras | *LS*, *GF*, *FC*, and *PH* | First sentence of the description of each taxon of each flora |

Note: *LS*, lifespan; *GF*, growth form; *FC*, flower color; *PH*, plant height.

**Appendix S2**. List of expressions representing approximations excluded prior to the extraction of plant height values.

**Expressions**

*c*

*c.*

*ca*

*ca.*

*ca.,*

*c.a.*

*cerca*

*about*

*aproximadamente*

*aprox.*

*approx.*

*approximately*

*aproximada*

*約*

*±*

Note: In cases where *de* was placed between the expressions listed above and a number (e.g., *ca de 1*), it was excluded from the search.

**Appendix S3**. Expressions excluded prior to the extraction of values for lifespan and growth form.

| **Database** | **Expression** | **Note** |
| --- | --- | --- |
| GBIF | *agicultura de temporal y cultivo anual* | Farming practice |
|  | *annual grassland* |  |
|  | *annual green tufts* |  |
|  | *annual high level cut* |  |
|  | *annual plant project* |  |
|  | *annual plot research* |  |
|  | *annual precipitation* |  |
|  | *annual prv visit* |  |
|  | *annual rainfall* |  |
|  | *annual report* |  |
|  | *annual stem* |  |
|  | *annual survey* |  |
|  | *annuals present* |  |
|  | *assemblage of mostly native annuals* |  |
|  | *assemblages of inner coast range perennial herbs and annual* |  |
|  | *bienal modal* | Farming practice (e.g., *campo sujo com queima bienal modal*) |
|  | *biennial cropping* |  |
|  | Combinations of {on/of/among/under} {a/the} {low/high} {tree/shrub} | Living environment (e.g., *on a high shrub*, *among the trees*, etc.) |
|  | *ex - herb.* | Abbreviation for herbarium |
|  | *ex herb.* | Abbreviation for herbarium |
|  | *ex- herb.* | Abbreviation for herbarium |
|  | *ex-herb.* | Abbreviation for herbarium |
|  | *ex. herb.* | Abbreviation for herbarium |
|  | *fire following annuals* |  |
|  | *grass accession* |  |
|  | *herbaceous sward occurs* |  |
|  | *in dirt and grass* |  |
|  | *lolium perenne* | species name |
|  | *mainly annual grasses* |  |
|  | *perennial bed* |  |
|  | *perennial dry pastures* |  |
|  | *perennial kale* |  |
|  | *perennial stream* |  |
|  | *pluviosidad anual* |  |
|  | *shrub tundra* |  |
|  | *tree bears fruit* |  |
|  | *voucher in herb* |  |
|  | *woody at* | Woodiness of the plant part |
|  | *woody base* | Woodiness of the plant part |
|  | *woody root* | Woodiness of the plant part |
|  | *woody-base* | Woodiness of the plant part |
|  | *woody-root* | Woodiness of the plant part |
|  | *一年生枝* | Current year branch (in Chinese) |
|  | *中文名:一年* | Part of species name (in Chinese) |
|  | *二年生枝* | Second year branch (in Chinese) |
| eFloras | Combinations of {*on*/*of*/*among*/*under*} and {*trees*/*shrubs*} | Living environment |
|  | *herb.* | Abbreviation for herbarium |
|  | *herbaria* |  |
|  | *herbarium* |  |
|  | *woody at base* |  |

**Appendix S4**. Expressions representing lifespan (*LS*) and growth form (*GF*) used to extract trait values from the text of GBIF and eFloras.

| **Database** | **Expression** | **Resolved class** |
| --- | --- | --- |
| GBIF | *anual* | Annual |
|  | *annual* | Annual |
|  | *annuelle* | Annual |
|  | *一年* | Annual |
|  | *biennial* | Annual |
|  | *bienal* | Annual |
|  | *二年* | Annual |
|  | *perennial* | Perennial |
|  | *perenne* | Perennial |
|  | *多年* | Perennial |
|  | *erva* | Herb |
|  | *forb* | Herb |
|  | *gras* | Herb |
|  | *grass* | Herb |
|  | *herb* (There is no period immediately after) | Herb |
|  | *herb.* (End of a field) | Herb |
|  | *herb. leaves* | Herb |
|  | *herb. flower* | Herb |
|  | *decumbent herb* | Herb |
|  | *weedy herb* | Herb |
|  | *prostrate herb* | Herb |
|  | *occasional herb* | Herb |
|  | *small herb* | Herb |
|  | *erect herb* | Herb |
|  | *endemic herb* | Herb |
|  | *rosetted herb* | Herb |
|  | *perennial herb* | Herb |
|  | *annual herb* | Herb |
|  | *terrestrial herb* | Herb |
|  | *rhizomatious herb* | Herb |
|  | *monocarpic herb* | Herb |
|  | *scapose herb* | Herb |
|  | *rooted herb* | Herb |
|  | *succulent herb* | Herb |
|  | *aquatic herb* | Herb |
|  | *abundant herb* | Herb |
|  | *branched herb* | Herb |
|  | *upright herb* | Herb |
|  | *epiphytic herb* | Herb |
|  | *slender herb* | Herb |
|  | *flowered herb* | Herb |
|  | *herbacea* | Herb |
|  | *herbaceo* | Herb |
|  | *herbaceous* | Herb |
|  | *herbe* | Herb |
|  | *hierba* | Herb |
|  | *sedge* | Herb |
|  | *weed* | Herb |
|  | *性狀:h* | Herb |
|  | *草本* | Herb |
|  | *草質藤本* | Herb |
|  | *abusto* | Wood |
|  | *arbiusto* | Wood |
|  | *arbol* | Wood |
|  | *arbolito* | Wood |
|  | *arborea* | Wood |
|  | *arboreo* | Wood |
|  | *arborescente* | Wood |
|  | *arbre* | Wood |
|  | *arbrisseau* | Wood |
|  | *arbusot* | Wood |
|  | *arbust* | Wood |
|  | *arbuste* | Wood |
|  | *arbustiva* | Wood |
|  | *arbustivo* | Wood |
|  | *arbusto* | Wood |
|  | *arvore* | Wood |
|  | *arvoreta* | Wood |
|  | *arvoreto* | Wood |
|  | *avore* | Wood |
|  | *avoreto* | Wood |
|  | *bark* | Wood |
|  | *baum* | Wood |
|  | *bush* | Wood |
|  | *liana* | Wood |
|  | *mallee* | Wood |
|  | *shrub* | Wood |
|  | *shurb* | Wood |
|  | *suffrutex* | Wood |
|  | *tree* | Wood |
|  | *woody* | Wood |
|  | *喬木* | Wood |
|  | *性狀:sh* | Wood |
|  | *性狀:t* | Wood |
|  | *木質藤本* | Wood |
|  | *木本* | Wood |
|  | *灌木* | Wood |
|  |  |  |
| eFloras | *annual* | Annual |
|  | *biennial* | Annual |
|  | *perennial* | Perennial |
|  | *herb* | Herb |
|  | *vine herbaceous* | Herb |
|  | *vine, herbaceous* | Herb |
|  | *climber, herbaceous* | Herb |
|  | *herbaceous climber* | Herb |
|  | *herbaceous vine* | Herb |
|  | *tree* | Wood |
|  | *shrub* | Wood |
|  | *liana* | Wood |
|  | *vine woody* | Wood |
|  | *vine, woody* | Wood |
|  | *climber woody* | Wood |
|  | *climber, woody* | Wood |
|  | *woody climber* | Wood |
|  | *woody vine* | Wood |

Italics denote the expressions used for the search without alterations

Expressions with the prefix *sub-* (e.g., subshrub) and suffix *-let* (e.g., shrublet) were included in the extraction.

Expressions are written in the singular, but the plural forms were also included in the extraction.

**Appendix S5**. Patterns of expression that could potentially cause false detection of plant height values that were removed from the records in GBIF and eFloras.

| **Order of expression, number, and unit** | **Category of expression** | **Expression** | **Note** |
| --- | --- | --- | --- |
| An expression is placed immediately **after** the combination of number and unit (e.g., *10 m width*). | Area, elevation, and location | *2* |  |
|  |  | *²* |  |
|  |  | *^2* |  |
|  |  | *across* |  |
|  |  | *above sea level* |  |
|  |  | *cell* |  |
|  | Length, diameter, and width | *diam** | When *in* or *de* was placed between a unit and an expression (e.g., “*1 m in width*”), they were both removed |
|  |  | *wide* |  |
|  |  | *dbh* |  |
|  |  | *d.b.h.* |  |
|  |  | *dap* |  |
|  |  | *long* |  |
|  |  | *largo* |  |
|  |  | *comprimento* |  |
|  |  | *width* |  |
|  |  | *tall at anthesis* |  |
|  | Other numerical data that may not be related to plant size (e.g., distance) | *o.* | Found in Swedish and Norwegian texts (e.g., “*73 m o.h.*”, “*vagkant vag z834 ca 40 m v om bro over hogan.*”) |
|  |  | *o h* |  |
|  |  | *.o.* |  |
|  |  | *o* |  |
|  |  | *nv* |  |
|  |  | *v* |  |
|  |  | *sv* |  |
| An expression is placed immediately **before** the combination of number and unit (e.g. *width 10 m*). | Length, diameter, and width | *width* | When *=* or *:* was placed between an expression and a number (e.g., *width = 1m*), they were removed together |
|  |  | *width to* |  |
|  |  | *width at* |  |
|  |  | *dbh of* |  |
|  |  | *dbh* |  |
|  |  | *d.b.h.* |  |
|  |  | *dap* |  |
|  |  | *diameter* |  |
|  |  | *diametro de* |  |
|  |  | *徑* |  |
|  |  | *長* |  |

When one of these expressions was placed immediately before or after the combination of number and unit (e.g., *10 m width*, *width 10 m*, etc.), the combination of the number, unit, and expression was removed.

Numbers included integers, decimals, fractions, and mixed fractions, and the units are listed in Appendix S7.

Combination of numbers and units can be within this range. An asterisk following an expression indicates all words starting from the expression were used.

**Appendix S6**. Patterns of expression used to extract values of plant height (*PH*) from GBIF and eFloras. The definitions of the numbers, units, and their combinations are the same as those in Appendix S5. However, values with more than three digits (e.g., *1000*) were ignored.

For GBIF, we used two expression patterns to extract *PH* values. The first pattern, which detects expressions such as *10m tree* or *10 m tall,* was

{Combination of numbers and units} {{*LS* or *GF*} or {*tall*/*high*/*alt*/*alt.*/*alltura*/*alto*/*altura*/*haut*/*in height*/*heigh*/*height*}},

where {*LS* or *GF*} represents *LS* or *GF* as shown in Appendix S4, and {*tall*/*high*/*alt*/*alt.*/*alltura*/*alto*/*altura*/*haut*/*in height*/*heigh*/*height*} are shown in parentheses.

The second pattern, which detected expressions such as *tree 10 m* or *height 10 m* was

{{*LS*, *GF*} or {whole plant body}} {*to*/*up to*/*de*/*ate*/*de ate*/*com*/*cerca de*/*de cerca de*/*com cerca de*/*of*/*:*/*,*} {Combination of number and unit},

where {*LS* or *GF*} is an expression representing *LS* or *GF* shown in Appendix S4, {whole plant body} is an expression representing the whole plant body shown in Appendix S8, and {*to*/*up to*/*de*/*ate*/*de ate*/*com*/*cerca de*/*de cerca de*/*com cerca de*/*of*/*:*/*,*} is one of the expressions in the brackets, which can also be missing (i.e., both *tree to 10 m* and *tree 10m* are possible).

For eFloras, the following pattern was used:

{*plant*/*culm*/*stem*/*herb*/*tree*/*shrub*/*vine*/*liana*/*climber*/*annual*/*biennial*/*perennial*} {Combination of numbers and units}

where the first bracket contains an expression.

**Appendix S7**. Units used for plant height extraction.

| **Unit** | **Resolved unit** |
| --- | --- |
| *m* | meter |
| *meter* |  |
| *metro* |  |
| *metre* |  |
| *met* |  |
| *mt* |  |
| *米* |  |
| *公尺* |  |
| *dm* | decimeter |
| *cm* | centimeter |
| *centimeter* |  |
| *厘米* |  |
| *公分* |  |
| *mm* | millimeter |
| *foot* | foot |
| *feet* |  |
| *ft* |  |
| *'* |  |
| *inch* | inch |
| *in* |  |
| *''* (two single-quotes) |  |
| *"* (one double-quote) |  |
| *""* (two double-quotes) |  |

Expressions are written in the singular, but the plural forms were also included in extractions.

For eFloras, only *mm*, *cm*, *dm*, and *m* were considered.

**Appendix S8**. Expressions representing the whole plant body for the extraction of *PH* from GBIF (see Appendix S6).

**Expression**

*alltura*

*alt*

*alt.*

*alto*

*altura*

*climber*

*climbing*

*de h*

*erect*

*ereta*

*escandente*

*fern*

*haut*

*hauteur*

*height*

*(hight)*

*(high)*

*ht*

*ht.*

*hgt*

*hgt.*

*h*

*plant*

*planta*

*stem*

*straight*

*succulent*

*twiner*

*twining*

*株高*

*體高*

*高度*

Expressions are written in the singular, but the plural forms were also included in extractions.

**Appendix S9**. Combinations of values in the *TraitNames*, *DataName*, and *OrigValueStr* columns used to assign candidate values of lifespan (*LS*) and growth form (*GF*) for each record in TRY.

| **Trait** | **Matching method** | **Corresponding values in columns** | | | **Resolved class** |
| --- | --- | --- | --- | --- | --- |
|  |  | ***TraitName* column** | ***DataName* column** | ***OrigValueStr* column** |  |
| *LS* | (a) | *Plant lifespan (longevity)* | Any | Numbers ≤ 2 | Annual |
|  |  |  | Any | Numbers > 2 | Perennial |
|  |  |  | Any | *poly-annuals* | Perennial |
|  |  |  | *Plant phenology: Annual* | ***yes*** | Annual |
|  |  |  | *Plant phenology: Biennial* | ***yes*** | Annual |
|  |  |  | *Plant lifespan: annual* | ***yes*** | Annual |
|  |  |  | *Plant phenology: Perennial* | ***yes*** | Perennial |
|  |  |  | *Plant lifespan: perennial* | ***yes*** | Perennial |
|  | (b) | *Plant lifespan (longevity)*, *Plant growth form*, *Plant life form (Raunkiaer life form)*, *Plant woodiness*, *Plant vegetative regeneration capacity*, *Plant functional type (PFT)* | Any | *annual* | Annual |
|  |  |  | Any | ***ann*** | Annual |
|  |  |  | Any | *biannual* | Annual |
|  |  |  | Any | *biennial* | Annual |
|  |  |  | Any | *biennual* | Annual |
|  |  |  | Any | *bieenial* | Annual |
|  |  |  | Any | *bennial* | Annual |
|  |  |  | Any | *perennial* ^a^ | Perennial |
|  |  |  | Any | *pernnial* | Perennial |
|  |  |  | Any | *pluriennial* | Perennial |
|  |  |  | Any | ***pere*** | Perennial |
|  |  |  | Any | ***1, 2, more*** | Perennial |
| *GF* | (b) | *Plant lifespan (longevity)*, *Plant growth form*, *Plant life form (Raunkiaer life form)*, *Plant woodiness*, *Plant vegetative regeneration capacity*, *Plant functional type (PFT)* | Any | *herb* | Herb |
|  |  |  | Any | ***h*** | Herb |
|  |  |  | Any | *forb* | Herb |
|  |  |  | Any | *herbaceous* | Herb |
|  |  |  | Any | *gras* | Herb |
|  |  |  | Any | *grass* | Herb |
|  |  |  | Any | *graminoid* | Herb |
|  |  |  | Any | *non-woody* | Herb |
|  |  |  | Any | *non woody* | Herb |
|  |  |  | Any | *false* | Herb |
|  |  |  | Any | tree | Wood |
|  |  |  | Any | ***t*** | Wood |
|  |  |  | Any | ***w*** | Wood |
|  |  |  | Any | ***s*** | Wood |
|  |  |  | Any | *shrub* | Wood |
|  |  |  | Any | *woody* ^b^ | Wood |
|  |  |  | Any | *liana* | Wood |
|  |  |  | Any | *palm* | Wood |
|  |  |  | Any | *bamboo* | Wood |
|  |  |  | Any | *mallee* | Wood |
|  |  |  | Any | *true* | Wood |

^a^ When it appeared as *not perennial*, the occurrence was ignored.

^b^ *Non woody* and *non-woody* were classified as herbs.

For the matching method (a), values in the three aligned columns in each row were matched, whereas all combinations of *TraitName* and *OrigValueStr* were considered for method (b).

Italics denote the expressions used for the search.

When assigning trait values, partial matches of expressions with cell contents were permitted, whereas expressions shown in bold italics were evaluated based on an exact match with the cell contents.

The expressions are written in the singular, but the plural forms were also included in the extraction.

**Appendix S10**. Combinations of values in the *trait_name* and *trait_value* columns used to assign candidate values of lifespan (*LS*) and growth form (*GF*) for each record in BIEN.

| **Trait** | **Matching method** | **Correspondent values in columns** | | **Resolved class** |
| --- | --- | --- | --- | --- |
|  |  | ***trait_name* column** | ***trait_value* column** |  |
| *LS* | (a) | *longest whole plant longevity*, *maximum whole plant longevity* | Numbers ≤ 2 | Annual |
|  |  |  | Numbers > 2 | Perennial |
|  | (b) | *whole plant growth form* | *pere* | Perennial |
| *GF* | (b) | *whole plant growth form*, *whole plant growth form diversity*, *whole plant woodiness* | *herb* | Herb |
|  |  |  | *forb* | Herb |
|  |  |  | *herbaceous* | Herb |
|  |  |  | *gras* | Herb |
|  |  |  | *grass* | Herb |
|  |  |  | *graminoid* | Herb |
|  |  |  | *non-woody* | Herb |
|  |  |  | *tree* | Wood |
|  |  |  | *shrub* | Wood |
|  |  |  | *woody* ^a^ | Wood |
|  |  |  | *liana* | Wood |
|  |  |  | *palm* | Wood |
|  |  |  | *bamboo* | Wood |

^a^ *non-woody* plants are regarded as herbs.

For matching method (a), all combinations of *trait_name* and *trait_value* were considered when assigning candidate trait values, whereas the values of the two columns aligned in each row were matched together for method (b).

Italics denote the expressions used for the search.

When assigning trait values, partial matches of expressions with cell contents were permitted.

**Appendix S11**. Number of unique and overlapping species when comparing GBIF with four other databases (the number of species shown in Figure 2).

| **Trait** | **Database(s) to compare** | **Threshold** | **Only in GBIF** | **Overlap** | **Only in other(s)** | **Total** |
| --- | --- | --- | --- | --- | --- | --- |
| Lifespan | All others | 0.5 | 38029 | 93866 | 109161 | 241056 |
| Lifespan | All others | 0.6 | 37567 | 92842 | 110185 | 240594 |
| Lifespan | All others | 0.7 | 37039 | 91574 | 111453 | 240066 |
| Lifespan | All others | 0.8 | 36667 | 90349 | 112678 | 239694 |
| Lifespan | All others | 0.9 | 36122 | 88328 | 114699 | 239149 |
| Lifespan | All others | 1 | 35561 | 84047 | 118980 | 238588 |
| Lifespan | TRY | 0.5 | 72764 | 59131 | 25911 | 157806 |
| Lifespan | TRY | 0.6 | 71776 | 58633 | 26409 | 156818 |
| Lifespan | TRY | 0.7 | 70630 | 57983 | 27059 | 155672 |
| Lifespan | TRY | 0.8 | 69728 | 57288 | 27754 | 154770 |
| Lifespan | TRY | 0.9 | 68282 | 56168 | 28874 | 153324 |
| Lifespan | TRY | 1 | 66389 | 53219 | 31823 | 151431 |
| Lifespan | BIEN | 0.5 | 95100 | 36795 | 10342 | 142237 |
| Lifespan | BIEN | 0.6 | 93686 | 36723 | 10414 | 140823 |
| Lifespan | BIEN | 0.7 | 91990 | 36623 | 10514 | 139127 |
| Lifespan | BIEN | 0.8 | 90491 | 36525 | 10612 | 137628 |
| Lifespan | BIEN | 0.9 | 88115 | 36335 | 10802 | 135252 |
| Lifespan | BIEN | 1 | 84430 | 35178 | 11959 | 131567 |
| Lifespan | eFloras | 0.5 | 116039 | 15856 | 14413 | 146308 |
| Lifespan | eFloras | 0.6 | 114857 | 15552 | 14717 | 145126 |
| Lifespan | eFloras | 0.7 | 113436 | 15177 | 15092 | 143705 |
| Lifespan | eFloras | 0.8 | 112216 | 14800 | 15469 | 142485 |
| Lifespan | eFloras | 0.9 | 110281 | 14169 | 16100 | 140550 |
| Lifespan | eFloras | 1 | 106874 | 12734 | 17535 | 137143 |
| Lifespan | Rice | 0.5 | 58570 | 73325 | 93530 | 225425 |
| Lifespan | Rice | 0.6 | 57852 | 72557 | 94298 | 224707 |
| Lifespan | Rice | 0.7 | 57020 | 71593 | 95262 | 223875 |
| Lifespan | Rice | 0.8 | 56350 | 70666 | 96189 | 223205 |
| Lifespan | Rice | 0.9 | 55421 | 69029 | 97826 | 222276 |
| Lifespan | Rice | 1 | 54201 | 65407 | 101448 | 221056 |
| Growth form | All others | 0.5 | 41166 | 108112 | 104194 | 253472 |
| Growth form | All others | 0.6 | 39844 | 104578 | 107728 | 252150 |
| Growth form | All others | 0.7 | 38416 | 100293 | 112013 | 250722 |
| Growth form | All others | 0.8 | 37358 | 96575 | 115731 | 249664 |
| Growth form | All others | 0.9 | 35782 | 90338 | 121968 | 248088 |
| Growth form | All others | 1 | 33858 | 77225 | 135081 | 246164 |
| Growth form | TRY | 0.5 | 62595 | 86683 | 71701 | 220979 |
| Growth form | TRY | 0.6 | 60788 | 83634 | 74750 | 219172 |
| Growth form | TRY | 0.7 | 58846 | 79863 | 78521 | 217230 |
| Growth form | TRY | 0.8 | 57396 | 76537 | 81847 | 215780 |
| Growth form | TRY | 0.9 | 55267 | 70853 | 87531 | 213651 |
| Growth form | TRY | 1 | 52621 | 58462 | 99922 | 211005 |
| Growth form | BIEN | 0.5 | 84510 | 64768 | 28561 | 177839 |
| Growth form | BIEN | 0.6 | 81692 | 62730 | 30599 | 175021 |
| Growth form | BIEN | 0.7 | 78557 | 60152 | 33177 | 171886 |
| Growth form | BIEN | 0.8 | 76183 | 57750 | 35579 | 169512 |
| Growth form | BIEN | 0.9 | 72567 | 53553 | 39776 | 165896 |
| Growth form | BIEN | 1 | 67761 | 43322 | 50007 | 161090 |
| Growth form | eFloras | 0.5 | 131943 | 17335 | 13527 | 162805 |
| Growth form | eFloras | 0.6 | 127553 | 16869 | 13993 | 158415 |
| Growth form | eFloras | 0.7 | 122395 | 16314 | 14548 | 153257 |
| Growth form | eFloras | 0.8 | 118160 | 15773 | 15089 | 149022 |
| Growth form | eFloras | 0.9 | 111413 | 14707 | 16155 | 142275 |
| Growth form | eFloras | 1 | 99362 | 11721 | 19141 | 130224 |
| Growth form | Rice | 0.5 | 72767 | 76511 | 62069 | 211347 |
| Growth form | Rice | 0.6 | 69768 | 74654 | 63926 | 208348 |
| Growth form | Rice | 0.7 | 66237 | 72472 | 66108 | 204817 |
| Growth form | Rice | 0.8 | 63521 | 70412 | 68168 | 202101 |
| Growth form | Rice | 0.9 | 59539 | 66581 | 71999 | 198119 |
| Growth form | Rice | 1 | 54494 | 56589 | 81991 | 193074 |
| Flower color | All others | 0 | 99101 | 12006 | 8416 | 119523 |
| Flower color | All others | 0.2 | 98707 | 11970 | 8452 | 119129 |
| Flower color | All others | 0.4 | 92864 | 10944 | 9478 | 113286 |
| Flower color | All others | 0.6 | 75913 | 8464 | 11958 | 96335 |
| Flower color | All others | 0.8 | 60477 | 6420 | 14002 | 80899 |
| Flower color | All others | 1 | 48564 | 4421 | 16001 | 68986 |
| Flower color | TRY | 0 | 104142 | 6965 | 3162 | 114269 |
| Flower color | TRY | 0.2 | 103732 | 6945 | 3182 | 113859 |
| Flower color | TRY | 0.4 | 97546 | 6262 | 3865 | 107673 |
| Flower color | TRY | 0.6 | 79741 | 4636 | 5491 | 89868 |
| Flower color | TRY | 0.8 | 63533 | 3364 | 6763 | 73660 |
| Flower color | TRY | 1 | 50830 | 2155 | 7972 | 60957 |
| Flower color | BIEN | 0 | 109081 | 2026 | 481 | 111588 |
| Flower color | BIEN | 0.2 | 108658 | 2019 | 488 | 111165 |
| Flower color | BIEN | 0.4 | 102018 | 1790 | 717 | 104525 |
| Flower color | BIEN | 0.6 | 83141 | 1236 | 1271 | 85648 |
| Flower color | BIEN | 0.8 | 66081 | 816 | 1691 | 68588 |
| Flower color | BIEN | 1 | 52628 | 357 | 2150 | 55135 |
| Flower color | eFloras | 0 | 105859 | 5248 | 5116 | 116223 |
| Flower color | eFloras | 0.2 | 105445 | 5232 | 5132 | 115809 |
| Flower color | eFloras | 0.4 | 98929 | 4879 | 5485 | 109293 |
| Flower color | eFloras | 0.6 | 80359 | 4018 | 6346 | 90723 |
| Flower color | eFloras | 0.8 | 63671 | 3226 | 7138 | 74035 |
| Flower color | eFloras | 1 | 50730 | 2255 | 8109 | 61094 |
| Maximum plant height | All others | 1 | 93709 | 31964 | 23190 | 148863 |
| Maximum plant height | All others | 2 | 70450 | 27435 | 27719 | 125604 |
| Maximum plant height | All others | 3 | 55744 | 23763 | 31391 | 110898 |
| Maximum plant height | All others | 4 | 47980 | 21801 | 33353 | 103134 |
| Maximum plant height | TRY | 1 | 103854 | 21819 | 9576 | 135249 |
| Maximum plant height | TRY | 2 | 78429 | 19456 | 11939 | 109824 |
| Maximum plant height | TRY | 3 | 61967 | 17540 | 13855 | 93362 |
| Maximum plant height | TRY | 4 | 53395 | 16386 | 15009 | 84790 |
| Maximum plant height | BIEN | 1 | 119393 | 6280 | 1616 | 127289 |
| Maximum plant height | BIEN | 2 | 92010 | 5875 | 2021 | 99906 |
| Maximum plant height | BIEN | 3 | 73975 | 5532 | 2364 | 81871 |
| Maximum plant height | BIEN | 4 | 64493 | 5288 | 2608 | 72389 |
| Maximum plant height | eFloras | 1 | 110662 | 15011 | 15212 | 140885 |
| Maximum plant height | eFloras | 2 | 85495 | 12390 | 17833 | 115718 |
| Maximum plant height | eFloras | 3 | 69354 | 10153 | 20070 | 99577 |
| Maximum plant height | eFloras | 4 | 60730 | 9051 | 21172 | 90953 |

The meaning of each field is the same as in Figure 2.

*Threshold*: Thresholds of criteria used for the removal of species from GBIF.

Criterion for *LS*, *GF*, and *FC* is the proportion of records with the most frequent value, and the criterion for *PH_max_* is the number of records with plant height.

Species with a criterion below the threshold are removed from GBIF.

Minimum threshold values (0.5 for lifespan and growth form, 0 for flower color, and 1 for maximum plant height) represent cases where no species were removed.

*Only in GBIF*: number of species unique to GBIF.

*Overlap*: number of species in GBIF and other database(s).

*Only in other database(s)*: number of species unique to other database(s).

*Total*: total number of these three groups.

*Overlap* also indicates the number of species used in the congruency calculation (Figure 3).
